# CrossFit and “Cancel Culture”: Probing Practitioners’ Responses to the “Canceling” of Greg Glassman

**DOI:** 10.1177/01937235231223986

**Published:** 2023-12-27

**Authors:** Dale Spencer, Derek Silva, Delphine DiTecco, Carmen West

**Affiliations:** 1 Department of Law and Legal Studies, 6339 Carleton University, Ottawa, Ontario, Canada; 2 King's University College at Western University, London, Ontario, Canada; 3 Department of Law and Legal Studies, 6339 Carleton University, Ottawa, Ontario, Canada; 4 Department of Sociology, 6339 Carleton University, Ottawa, Ontario, Canada

**Keywords:** Cancel culture, crossfit, racism, Greg Glassman, sport communities

## Abstract

In this article, we explore the responses of crossfit practitioners to the ‘canceling’ of Greg Glassman in the aftermath of racist tweets and comments made in response to the killing of George Floyd. We draw on 50 interviews with crossfit practitioners to understand how they interpret and respond to the ‘canceling’ of Greg Glassman and the disavowal of CrossFit by prominent CrossFit athletes and organizations. We probe how athletes, regardless of levels of involvement, in the wake of Glassman's comments respond to the refiguring of the sporting community of CrossFit. A cancel culture continuum from affirmation to rejection emerged from the interview data that typified their views of cancel culture, Greg Glassman's removal from CrossFit HQ, and the current state of the sport. We conclude with a discussion of the phenomena of canceling or cancel culture and reflects on crossfit as a sport in light of the Glassman affair.

## Introduction

On June 6, 2020, in response to a Tweet from the Institute for Health Metrics and Evaluation at the University of Washington stating that “racism and discrimination are critical public health issues that demand an urgent response,” founder of and then-CEO of CrossFit Greg Glassman responded “It's FLOYD-19,” prompting an immense backlash on the social media platform and in mainstream media (Maida et al., 2020).^1^ Earlier that day, Glassman hosted a Zoom call with CrossFit gym owners where he promulgated conspiracy theories about COVID-19 and claimed that George Floyd, a 46-year-old Black man who was killed by police on May 25, 2020, had been killed as part of the extensive cover-up of counterfeiting and unrelated to racism (Brooks & Mack, 2020).

The response by prominent members in the crossfit community to Glassman's Tweets and statements was nothing less than volcanic (Brooks & Mack, 2020). Despite a long history of underrepresentation of Black, Indigenous, and People of Color (particularly racialized women) within crossfit spaces (Sanchez, 2019; Walsh, 2020), prominent CrossFit Games athletes denounced Glassman and broke off from CrossFit games, related competitions, and even the brand itself (Maida et al., 2020). CrossFit-affiliated gyms across the globe reacted by ending their affiliation with CrossFit, the brand's most notable sponsor, Reebok, ended their corporate association with CrossFit (Lim, 2020), and numerous other brands denounced his statements publicly. Subsequently, Glassman pleaded in a statement released by CrossFit's Twitter that his statements were a mistake but that they were not racist (CrossFit, 2020). On June 9th, 3 days after Glassman's Tweet, he resigned as CEO. In addition, facing considerable pressure both within CrossFit HQ and by affiliates, 2 weeks later announced he was putting the company up for sale. Glassman had been, as some put it, ‘canceled’ (Anderson, 2020).

Considerable debate came in the wake of Glassman leaving CrossFit HQ and the mass deaffiliations with the CrossFit brand. In addition, the removal of Glassman and repudiation of CrossFit HQ was viewed, by some prominent figures in the sport, as another example of so-called “cancel culture” (Anderson, 2020). We broadly define cancel culture as the mass disengagement, disavowal, or exclusion of people or groups in public and or private settings for the breakage of social norms (Clark, 2020; Ng, 2020; Norris, 2023). Regardless of one's viewpoint within the sport, the disaffiliation movement had a significant impact on gyms, crossfit practitioners, and the sport of crossfit itself.

This article probes how CrossFit practitioners interpret and respond to the “canceling” of Greg Glassman and the disavowal of CrossFit by prominent CrossFit athletes and organizations. We are concerned with how athletes, regardless of levels of involvement, in the wake of Glassman's comments respond to the refiguring of the sporting community of CrossFit. Whereas there is an emerging literature on the phenomenon of “cancel culture,” there remains a dearth of research on responses to “cancel culture” within subcultures such as sporting communities. Beyond the relative paucity of focus on responses to “cancel culture,” rather than offer a full etymological discussion about the definitional characteristics of “cancel culture,” our goal here is to contextualize the nuances of individual and group *responses* to events perceived to be “cancel culture” by participants. Drawing on 50 interviews with crossfit practitioners, we examine their interpretations of disaffiliation, how they view the sport now, and their view of the impact of “cancel culture”—the Glassman affair—on their sport.

We structure this article in three main sections. First, we offer an overview of the literature on cancel culture and its relevance to this study. We then describe CrossFit—both the brand and the sport—and describe the methods used. Third, and last, we analyze the results of our study before engaging in a discussion of the relevance of our findings to both our understanding of so-called cancel culture in communal sporting spaces, but also how we might interpret these findings to broader social phenomena.

## What Is “Cancel Culture”?

The recent phenomenon of individuals being “canceled” on the Internet has piqued the interest of popular media and scholars alike. The phenomenon that has been termed “cancel culture” can be explained as an Internet practice, where individuals perceived to have broken societal norms are condemned on social and other media platforms (Ng, 2020). There is a wide range of disputes on how cancel culture was created and termed, with some arguing that the phenomenon was created in conjunction with the #MeToo movement (Greenspan, 2020; Holman, 2020), and others convincingly suggesting it has roots in Black vernacular tradition which has been misappropriated by hegemonic groups in contemporary culture to serve the needs of social elites (Brock, 2020; Clark, 2015, 2020; Nakamura, 2015).

The origin of cancel culture and its relation to other terminology is disputed, however, there is agreement on the broad description of cancel culture. Cancel culture is described as the collective voices of individuals—typically marginalized individuals—that attempt to “call-out” or express their disagreement with individuals’ behaviors or views (Ng, 2020). Following the call-out, individuals usually experience a withdrawal of support from the collective, which can include the retraction of both monetary and temporal support. Individuals who have experienced this call-out and subsequent lack of support are considered to have been canceled (Clevenger, 2019; Ng, 2020). The purpose of an individual being canceled is that the collective can communicate supposedly evolved social norms while condemning individuals that break them (Holman, 2020). The condemnation of the individual and the continual withdrawal of support then force them to consider their actions and accept responsibility in the hopes of regaining support (Clevenger, 2019).

The first element of cancel culture is what is described as the boycott or withdrawal of support that occurs. For Brito (2021), “it is the withdrawal of financial support, political support, social, economic support, often in pop culture in the form of attention of a particular media star, a political figure, a business figure.” The withdrawal of support is done to inform others that they should similarly withdraw their support. The second element is the silencing of the individual, which occurs through the lack of support and attention (Brito, 2021). This collective silencing is thought to result in social isolation, loss of status, and/or reduction in social or economic capital for those being ‘canceled.’

Cancel culture, is comprised of two separate and distinct groups of actors: those who have been “canceled” and those who contribute to, participate in, or otherwise support the so-called cancel. Cancel culture has been described as a form of norm enforcement consisting of norm violators and norm enforcers (Norris, 2023). As outlined by Meredith Clark (2015, 2020) and others (Brock, 2020; Nakamura, 2015), the “callout” on social media platforms is a form of activism that originated in racialized and femininized communities to enforce ever-evolving social norms around social justice, equity, diversity, and inclusion. Indeed, when cancel culture first emerged as a cultural artifact, the norm enforcers were typically racialized and feminized actors seeking swift and widespread accountability for legitimate social harms. Individuals attempted to reclaim their power, voice, and agency in society by fighting against the norms that they believed aided their oppression. The norm enforcers called out the norm violators who were typically individuals in positions of power or held privilege (Ng, 2020; Semiramis, 2019). Cancel culture has since evolved—or as Clark (2015, 2020) maintains, misappropriated by social elites—to a point where norm enforcers include both marginalized and hegemonic individuals and groups (Ng, 2020; Semiramis, 2019). Importantly, research has found that individuals who are norm enforcers are more likely to be socially responsible and open to new experiences (Skoric et al., 2010). Additionally, it was found that individuals who were socially responsible, agreeable, and neurotic were more likely to be deterred by the risk of cancelation. Therefore, individuals who were considered socially responsible were more likely to contribute or cooperate with cancel culture (Skoric et al., 2010).

The #MeToo Movement is widely believed to have brought cancel culture to the forefront of public attention by calling out a general indifference toward the harms of sexual harassment and assault, creating a method for individuals to come forward with allegations and the subsequent canceling of the individual who had allegations leveled against them (Holman, 2020). Cancel culture is considered to have evolved in many ways during its short existence. For instance, cancel culture developed as a way of holding individuals accused of sexual harassment and assault accountable and creating a culture that no longer allows sexual harassment and assault to be swept under the rug. It has since broadened to include individuals being canceled for a plethora of reasons, although the cancelation is frequently related to a perceived norm violation (Holman, 2020). Another evolution of cancel culture is in relation to the temporal dimension. Here norm enforces have sought the inclusion of historic norm violations in their pursuit of accountability (Brito, 2021). An example of this retroactive cancelation is Kevin Hart, who was canceled for what were considered to be homophobic Tweets that he had posted 10 years prior. Hart faced many repercussions due to his cancelation, including immense pressure to step down from his role as host at the 2019 Oscars (BBC, 2019).

Many individuals equate the mob of people that accumulates when an individual is canceled to a crowd of people with pitchforks in the medieval period (Semiramis, 2019). People who disagree with cancel culture suggest that norm violations should be met with empathy and education in order to create an environment of forgiveness and growth. Without the ability to properly understand their wrongdoing, how to avoid a norm violation moving forward, as well as the violated norm itself, norm violators are denied the opportunity for growth (Semiramis, 2019; Pohjonen & Udupa, 2017). Further, as André Brock (2020, p. 220) argues, the origins of a “cancel culture” among Black Twitter is often mistaken for such a “mob mentality” that the original practice of “signifyin,” or calling out, among Black women on social media is often an explicit “critique of systemic inequality rather than an attack against specific, individualistic transgressions.” Correspondingly, it is perhaps both misleading and inaccurate to reduce the complex processes that make up the phenomenon of canceling to merely mob groupthink. Another major criticism of cancel culture is that it limits individual freedom of expression while stopping the exchange of ideas and views, as well as limiting individuals’ interest to venture out of their comfort zones (Brito, 2021). The perceived limitation of free speech has led to cancel culture being viewed as a device to execute an ideological purge (Velasco, 2020). Cancel culture is further criticized as classifying individuals as good or evil, based solely on whether an individual has violated social norms or not. Following an individual's evil classification, there is a similar punishment for all transgressions, regardless of the severity of the individual’s actions (Brito, 2021; Brooks, 2022).

These criticisms of cancel culture notwithstanding, little empirical research has been conducted specifically on responses to cancel culture—in particular, how people respond to the canceling of key figures in their interest groups. Additionally, sport has been a prime site where so-called cancel culture has taken place and promulgated, and even less research has been conducted on the topic in this area. As such, this article attempts to gain a deeper understanding of how people respond to the so-called canceling of important figures within recreational spaces. Taking the “canceling” of Greg Glassman and crossfit as our case study, this study probes how practitioners within the sport make sense of and respond to cancel culture.

## What Is CrossFit?

Crossfit as an activity is one of the fastest-growing high-intensity activities in the world (Claudino et al., 2018). As a form of high-intensity functional training, its practitioners use elements of gymnastics, weightlifting, powerlifting, plyometrics, and cardiovascular activities together to complete functional movements called “workouts of the day.” These are completed in gyms or “boxes,” where all the necessary equipment (i.e., dumbbells, weights, ropes, bands, or racks) are available. Practitioners may or may not train for crossfit competitions that are held at the local, national, or international levels. Crossfit must be distinguished from CrossFit as a brand. CrossFit is a brand associated with the functional movements of crossfit as an activity. The method and brand were developed by Greg Glassman, who founded and trademarked the brand with Lauren Jenai in 2000. CrossFit licenses the “CrossFit” name to gyms globally for an annual fee, certifies “CrossFit” trainers, offers training courses, and outlines best practices and codes of conduct for those associated with the “CrossFit” brand. While CrossFit and crossfit are inherently connected, they are increasingly autonomous and independent, particularly following the Greg Glassman case being studied here. This is to say that not all gyms are affiliated with CrossFit the brand and a practitioner can be doing crossfit without being at an affiliated box.

Crossfit participants have been described as having an evangelical zeal (Beck, 2017) for their sport, while some even refer to it as a “cult”-like environment (Dawson, 2015; Denby, 2013). Crossfit participants are often marked by identification with crossfit as part of their identity, personal transformations through practice, both physically and mentally, and a strong commitment to their local community (Dawson, 2015). Concomitantly, as a sport that consists of the combination of high-intensity interval training, powerlifting, gymnastics, Olympic weightlifting, and other exercises, CrossFit involves high levels of physical interaction between participants, competition between participants and oneself (previous times and weights of a given lift), and “loyalty to the box,” that is, cohesion with and loyalty to a participant's gym.

To date, much of the scholarly literature on crossfit focuses on representations of CrossFit in various forms of media or in the intersectional experiences of CrossFit participants. This body of work addresses key issues related to the intersectional modalities of inequality that are represented and often reified in crossfit cultures. Couture (2019), for example, draws on a critical discourse analysis of *CrossFit Kids Magazine* to illuminate the ways in which children are represented through the duality of “at-risk” subjects but also as “in-progress” future citizens, resulting in the promotion of active children and proactive parenting based primarily in individual risk management. Knapp (2015a, 2015b) illuminates how ideal femininity and hegemonic masculinity are at once reified but also resisted in the *CrossFit Journal* and in the gym. Similarly, Sanchez (2019) and others (Brown, 2015) importantly address the unique experiences of racialized women in CrossFit and other fitness spaces, highlighting how participants often feel like outsider “in the box” and are more likely to accept the unnecessary emotional labor associated with navigating cultural differences with mostly white participants (Denby, 2013). As a predominantly White and male space, crossfit often disregards the impact that its practices and figures have on racialized or otherwise marginalized folks and how ethnicity, gender, sexuality, and race intersect with the sport and its spaces (Brown, 2015; Denby, 2013; Sanchez, 2019; Washington & Economides, 2016). For this reason, crossfit is also an important space for the study of practitioner responses to incidents involving racist remarks from key figures in the sport. To this end, the present study seeks to explicitly examine the case of Greg Glassman in a relatively homogenous environment underrepresented by racialized and femininized participants.

Departing from previous research that looks at the inner workings of the sport and participation, in this article we focus on the CrossFit brand and affiliation and focus on practitioners’ conceptions of cancel culture and Greg Glassman's comments on George Floyd in the wake of his death outlined in the introduction of this article. We are not committed to the reality of cancel culture (as a “real” phenomenon or not), but interested in how crossfit practitioners, deeply committed to the sport, interpret Greg Glassman's removal as CEO of CrossFit HQ, his role in the sport and its development, and their view of how the affair was handled by CrossFit HQ, individual clubs across the globe, and their club. To be clear, we are foremost interested in their conceptions of “cancel culture” and the removal of Greg Glassman. To this end, we probe a “cancel culture continuum” reflecting the range of responses to Greg Glassman and his cancelation. In the next section, we outline our method of this study.

## Method

The research team conducted fifty 40–90 minute semistructured interviews with crossfit practitioners recruited from the province of Ontario, Canada (participants in the final sample resided in Ottawa, Toronto, and London, three cities in Ontario, Canada). Participants were recruited via advertisements posted on Facebook and Instagram, as well as through word-of-mouth and snowball sampling. Our inclusion criteria for interviews required recruits to be a current member at a CrossFit club and to have practiced for at least 6 months. The minimum time in the sport was 9 months, with the longest practicing for 14 years. The average participation was 5.5 years. The youngest participant was 23 years of age and the oldest was 62 years old; the average years of age was 42. Twenty-nine participants were parents and 48 were employed at the time of the interview; two were retired. Out of 50 participants, 33 identified as “woman” or “female” and 17 identified as “man” or “male.” When asked if they identified with an ethnic background, 28 participants reported identifying as White or Caucasian; eight as Western European; seven as Canadian; four as Latin American, Hispanic, or Mexican; three as Black; one as Asian and one as a visible minority (some participants identified with two or more ethnic backgrounds). Eight participants did not report identifying with an identifiable ethnic background.

Our exploration of cancel culture in the context of crossfit is part of a larger research project on the experiences of crossfit practitioners. Thus, topics covered in the interviews were intentionally wide-ranging: from views of the sport, motivations for participation, experiences as a practitioner, salience of community, impact of COVID-19, and views of Greg Glassman and the following decisions of CrossFit HQ. Questions were developed with the goal of understanding the experience of being a crossfit practitioner from a holistic perspective and to best address specific research questions, namely on the impact of important sociocultural events on this particular community (such as the canceling of Greg Glassman and the COVID-19 pandemic). Interview questions were developed with the help of a research assistant who practices crossfit themselves. This helped ensure the use of culturally relevant language and the development of questions that would best capture the crossfit practitioner experience. An interpretive-hermeneutic methodological approach (Denzin, 1989; Freeman, 2011; Schwandt, 1998) was used to analyze the interviews. In this approach, it is understood that each practitioner offers distinct interpretations of any given event, case, or of other members (Czarniawska, 1998). A qualitative approach best suits this particular study given the experiential nature of the intervention's focus on individual responses to perceived events of “cancel culture.” Given that both individual responses and the perception of so-called “cancel culture” will be diverse across practitioners, a qualitative, interview-based study is perhaps the only useful method to gain an understanding of the nuance and depth of individual participants' interpretations. The transcribed interviews were analyzed by multiple investigators, which entailed employing interpretive hermeneutics as an analytical approach and QSR NVivo as a qualitative data management and analysis software. Interpretive hermeneutics is an apposite approach as it is a methodological device for understanding how actors *interpret* the meanings of social actions and their respective worlds (Schwandt, 1998, 1999). In terms of process, a series of interpret themes were identified in the first five interviews by a single member of the team—and developed into a code book—and two additional project members used the code book to guide the coding of the remaining interview transcripts. QSR NVivo then aided in organizing the interpretive themes; the ‘cancel culture’ theme is analyzed below.

## Results—The Cancel Culture Continuum

A range of interpretations emerged in response to the questions: what do you think of Greg Glassman being canceled? What do you think about disaffiliation from CrossFit? These interpretations can be placed along a continuum of cancel culture, from affirmation to opposition to his firing and the reactions of the broader crossfit community. Such interpretations were in reference to Greg Glassman's statements, the reactions—by gyms, professional athletes, and amateur practitioners—toward Greg Glassman's “cancelation,” removal, or disavowal from crossfit, and their view of the phenomenon of “cancel culture” more generally in the political milieu. Respondent quotes presented in the following pages represent examples of the cancel culture continuum we outline here but are by no means the only examples.

The most common response to Greg Glassman is affirmation of his removal from CEO of CrossFit HQ. In other words, respondents had varying levels of endorsement for Glassman's removal as CEO and disavowal from crossfit communities. When asked how they viewed the reactions to Glassman's removal, David and Julia responded in the following way:

*Julia* (White, 47, teacher): Um … yeah like I said I guess it really doesn't—us going to our gym at the end of the day it doesn't really impact us but, but we didn't really want to be part of something that was clearly pretty wrong.

*David* (White, 57, warehouse worker): I don’t know if I thought it was fair right away because it's so I, you know, but after the more I read, the more I did my own research and kind of—absolutely I think uh—I think his he had he seemingly had a bit of an ego so I was a bit surprised that he let go of the company as easily as he did … I think getting him out was the right thing to do.

Both David and Julia represent a simplified affirmation of Glassman’s removal from the CrossFit world. For Julia, affiliation simply did not align with the broader views of her club, but disaffiliation will not affect the goings on of her club. Here, Julia articulates a preformed disaffiliation from not only the views of Glassman among members of her club but perhaps even a distancing between her club and CrossFit HQ more generally. For David, he views Glassman's removal as both surprising based on his understanding of the former CEO's sense of self and him leaving as “the right thing to do.” While simultaneously presenting surprise that Glassman decided to resign as CEO, David sketches out his own moral and ethical perspective, expressing that Glassman had, for him, violated his morality and thus deserved to be let go. This moral signposting was common amongst our participants. For example, Emma (white, 51, fitness instructor) decidedly articulated her perspective by responding to a question about her view on Glassman's comments by saying: “his comments don’t surprise me … because of who he is. What surprised me is the people who were very high up in the organization—how they said nothing for many years.”

In other cases, practitioners considered the removal of Glassman moved beyond the right thing to do to be a necessity for the sport. Consider the following responses:

*Bryan* (White, 44, administrator): To that. I definitely I think that's great, and then that forced to kind of change of ownership right. If … people are going to start dropping or high-level competitors are not going to compete, then yeah, I think that's they're using their platform properly and pushing for things like that that we really shouldn't be standing for but, on an individual level, I don't think you know me deciding well I’m not going to participate in CrossFit well that only affects my local gym.

*Elouise* (White, 47, teacher): So many components of cancel culture are just, like they need to be happening, they need to be shutting things down that has systematically excluded people and groups. And I’m okay with that, I’m okay with all the #Metoos and the increased opportunity for people to speak about their experiences.

*Roger* (White, 36, IT): I don't understand why disagreeing so quickly needs to be tied up into a buzzword like cancel culture, because the term of cancel culture puts a negative connotation on what it is people are doing. I totally agree with the side of the gyms to, to part ways if they don't agree with Glassman because it wasn't just a mistake that happened years ago, that could be forgivable. It was something that was very recent it was a very deliberate and it was something that wasn't followed up with an apology or a statement or anything like that. So, you know you can call it cancel culture, but at the end of the day, like this was a very true current and deliberate perspective from somebody who was at the top of CrossFit, so you have to you have to take a stance.

In both responses, the “canceling” of particular individuals is a necessity and a change that needed to happen. In relation to Bryan's response, it was a necessity to remove Glassman to sustain the sport of crossfit and CrossFit HQ, as a brand and corporation. The potential of disaffiliation is not just at the level of professional crossfit athletes but is also in relation to choices made by practitioners at the local level. For Elouise, the phenomenon of cancel culture writ large is justifiable as a way for individuals to speak out against institutions and individuals that have subjected marginalized groups to violence and/or injustice. Roger's response points to the fact that Glassman doubled down on his statements and that his statements were intended and well-thought-out. He believes that the only way in which to correct the orientation of CrossFit HQ is for gyms to disaffiliate. He also believes that the term cancel culture does a disservice to the people who are trying to correct a perceived wrongdoing. In some cases, participants went beyond the necessity of the implications of so-called cancel culture to recognize the need for accountability of key figures. Consider the following response:

*Kenny* (white, 46, paralegal): There is a need … I think … to make sure people are accountable for their actions. Like, people need to realize they can’t go around saying bad things that alienate or discriminate against people. Saying ‘Floyd-19’ right after an African-American was shot and killed and equating it with a virus that has killed more minorities at a higher rate? C’mon, man. Really? He needs to be responsible for saying that. He represents a big organization, and he needs to choose his words more wisely … and if he doesn’t there should be some sort of consequences. The world is changing all around us. Some of the things that were fine to say when I was growing up aren't any more. Some people don’t seem to get that … right? I have heard a lot of people use the term ‘accountability culture’ on social media and that makes more sense to me.

Kenny reflects on broader shifts in sensibilities that are part and parcel of civilizing spurts in Western society (Buschendorf et al., 2011; Elias, 2000). Whereas these shifts reflect shame and repugnance in relation to behaviors within the broader culture, on an individual level such sensitivities are not reflected universally. Accountability, in this sense, reflects a correction to views that are responded to with shame and repugnance. Kenny also evinces that Glassman represents the broader CrossFit organization and affiliates and his “accountability” represents a repudiation of his expressed views that do not align with the crossfit sports culture. Whereas close to half the respondents affirmed the dismissal of Glassman, a portion of the respondents accepted the reality of “cancel culture” but rejected Glassman's treatment in the aftermath of his statements.

While some participants affirmed cancel culture, others represented positions on the other end of the continuum by rejecting “cancel culture” to varying degrees. This implies the repudiation, dismissal, or renunciation of “cancel culture” as either a useful or valid concept or a social phenomenon altogether.

*Carlos* (Asian, 38, occupation unknown): I think we have to start taking things like not at face value, and I think that we have to do our own research and not just automatically all right cancel something or someone just because of something that they said or did. I think there has, like we like we as individuals have to do our own kind of due diligence and educate ourselves on you know kind of the scenario of what happened or you know why they did or said what they did. So I don't it's, not that I, I guess, I don't know I really necessarily don't agree with it, but it's, not that I don't either I think we just you know for me she's making sure that I’m fully educated on what happened. And not just saying ‘hey this person's like canceled’.

*Laura* (White, 45, lawyer): I feel like we're becoming a culture of people who just want to lash out at people and are waiting for a reason for people to fail and you know, everybody fails everybody makes mistakes, and if we're at the point where we expect perfection, then we better expect it from ourselves and God forbid, you make a mistake, because you know people will turn around and hold you to that standard as well and it's not attainable. I am a strong believer that we have to learn from history and by canceling the history we're basically saying it never happened, or we're not going to acknowledge that well, then we don't learn we don't learn from it we're not allowed to take those lessons that we, you know that we can learn and say okay, we don't agree with what things were what happened, back then, they were operating on the information that they had at the time. We know so much more now so we think that that's inappropriate and that's okay too here's how we're going to do better. So, my focus in life is about problem solving and what can we do to do better, and I think cancel culture is the direct opposite of that it's about smacking people down and personally attacking people without actually allowing people to do better and learn from their mistakes.

*Noah* (White, 36, professor): I’m not a fan (of cancel culture). I think having, you know, conversations about what's happening is important and being balanced and listening to the other side and changing your mind when new fact arise, or when a position is put out that maybe you didn't agree with or didn't consider initially. I think it's really important to to change your mind … but the idea of kind of an angry mob coming in and, you know, maybe being right, maybe not being right, but resulting in somebody losing a business or somebody really being harmed. I am not a fan.

Whereas these respondents do not condone the statements of Greg Glassman, they are on the opposite end of the cancel culture continuum. They are concerned with the implications of the phenomenon of cancel culture in terms of making mistakes and loss of jobs and businesses. Jason echoed Laura and Noah's depiction by expressing his condemnation of a “mob mentality” he interprets as a foundational characteristic of cancel culture:

*Jason* (White, 34, naturopath): The mob mentality on social media is the worst aspect of it. Again, it's more complex than people think. On one hand people need to be responsible for their actions and for what they say. On the other hand, people make mistakes. Does someone deserve to lose their livelihood because of a mistake? I don’t think so. We need to figure out a way to hold people responsible, but also not gang up on them until they get fired. Everyone makes mistakes and I bet I could go through the history of anyone and find something dumb they have done and blow it up on social media. So, in that way cancel culture is actually really scary … like maybe it could happen to anyone. I think it is a fine line between holding people responsible and also letting them atone for their mistakes. The whole mob mentality happened to Greg on social media and people ganged up on him until he quit or whatever. I think that is a bit far. But it is life now. Social media has that power and people need to be careful about what they say on it because it can be misconstrued and taken out of context. I guess I think we need to stop with the whole mob mentality on social media when someone does something dumb.

In a similar way, Tony went on to explain the perceived limitations and problems with the idea of “cancel culture” and how it does not adequately deal with the “nuance” of social life:

*Tony* (White, 43, occupation unknown): I think it's gone. Like, you know, like a lot of things, you know, they kind of go you go too far. It'll, I think, like, cancel culture will be canceled. At some point. Yeah, I'm not I'm not a huge fan, like, you know, history and, you know, everything. Everything's in the gray and you know … cancel culture does not do nuance in life which is about nuance and balance … I don't think by definition cancel culture can ever be like, you know, on point, it might be all that all the time, you know, it gets things right sometimes, like certain people deserve to be canceled, but certainly others don't. And it's unfortunate that people can't necessarily speak their mind without worrying about it.

*Charlotte* (White, 56, crossfit gym owner and coach): I don’t have that much to say, being kind of affiliated with CrossFit HQ other than a sort of more company line. We here at (name of club) CrossFit do not condone or otherwise support his comments in any way, and we strive to be a welcoming and inclusive environment for all. I know you probably want to ask a lot about Greg Glassman, but I think it is best if I don’t really answer those questions.

Noah went on to discuss how Glassman's comments changed his relationship with CrossFit the brand but, importantly, did not change his relationship with crossfit the sport.

*Interviewer:* Did your view of crossfit change at all after Glassman's comments?

*Noah:* Um my view of the brand did, my view of the kind of concept behind training did not. I would make that distinction.

Similarly, Isaac expressed his distaste for “cancel culture” by suggesting that the phenomenon is particularly “toxic”:

*Isaac* (Black, 49, banking): I would lean more toward not liking cancel culture because it's so toxic … I find everybody so quick to react and it's scary. To know that people's careers and livelihoods can be taken away because of like, you know, a false mistake … I’m terrified that I’ve been on Facebook, I get memories that pop that I posted before. I’m like, Jesus, I put that out there and I’m just a regular person, so like to be a celebrity or something and to get canceled for something you said at the moment you believed was funny. If you’re willing to apologize for it now, then I think you just accept the apology and you move on right? People grow, people change.

In doing so, Isaac, like Jason, expressed a common fear among respondents who reject the notion of cancel culture: fear of being canceled for past behavior and the perceived injustice of this happening to others. This fear then presented as justification for the rejection of the practices of disavowal that typically accompany “cancel culture.” Such views along the continuum evince a sense that mistakes can and will happen and there should be space for forgiveness. That said, none of the participants were on the extreme end of the continuum thereby committing to the idea that Glassman should be reinstated, or his views should be accepted.

Although an outright denial of the existence of cancel culture can be, in some ways, similar to rejection or ignorance, it is distinguished by the understanding of the importance of accountability for actions. While those expressing denial may disassociate from the concept of “cancel culture,” they tend to articulate the necessity of holding individuals accountable for their actions, which is something those who reject it altogether do not tend to do. Take Elijah and Jane's responses, for example:

*Elijah* (Black, 29, warehouse general manager): So what's your view of cancel culture? Participant: I don’t have too much thought about it. Say stupid things and face stupid consequences. To me it is more about people being held responsible for what they say. People are being held to a different standard than in the past, and I wouldn’t call that cancel culture. Cancel culture, to me, is more about being canceled or whatever for no reason. Not when people legitimately say hurtful things and have to face consequences. Yeah … I think that is my view.

*Jane* (White, 41, military): I feel the same way about the words cancel culture as I do about woke; I think that it's I think it's entirely fabricated it's just a word that people are using when they're when they're being held accountable. As far as I’m concerned, the only reason a whole bunch of people are holding you accountable is because it can be now broadcast to a greater audience with social media 24-h news cycle. Whereas before you know, even if you did something at school it's only the ears that it fell on, you know that, and people would judge you. You know and even then, it was probably hearsay to at least now, you can see, somebody's words, you can you know you can see everything so, I think that um he was just held accountable for what he said, especially since he doubled down on it afterward.

While not rejecting the effects of removing of Glassman, these respondents offer a more nuanced view along the cancel culture continuum. They can be placed alongside the affirmative end of the continuum for emphasizing responsibility for affronts to social norms, but they either do not like the term cancel culture or reject what happened to Glassman as cancel culture. Jerome, self-identifying as Hispanic and gay, spoke about his lack of specific interest in CrossFit HQ and Glassman, but did highlight his thoughts on some of the problems associated with the notion of “cancel culture”:

*Jerome*: I've never really been a huge CrossFit HQ guy. I don't really follow that news. I like CrossFit. I like the sport. I like my gym. But I don't really look at the big figures in the sport outside of Instagram and YouTube. You know training purposes … But when I heard about Greg Glassman's comments, I was not shocked there are a lot of people in CrossFit who share those beliefs, it's not shocking that that goes all the way to the top. I'm glad he resigned. I'm glad he was … let's call it canceled … although I don't believe in that cancel culture shit, I am much more aligned with the people who call it ‘accountability culture’ and you can't say shit like that if you are in a position of power. Hell, you can't even say that stuff if you're not. I faced a lot of discrimination in my life, and I try to not do that in my own life so in someone send an appropriate comment about Black or Indigenous groups I … I don't like that … he absolutely shouldn’t be the face of the sport if he's going to say shit like that. I’m a Gay man and I believe Black Lives Matter.

Jerome goes on to convey a more nuanced view of so-called “cancel culture” as being much more aligned with a culture of accountability for one's actions:

*Jerome:* To me, it's not cancel culture. You don't get canceled per se … you're held accountable for your actions. It's not your right to go spew racist or bigoted things to whoever you want without repercussion. So, when you say things that are harmful you need to be accountable. Social media provides that accountability so while some folks might not like it it's a reality of our lives. I don't post things on social media that I think are inappropriate. I don't say things in public that are harmful and if you do I think you need to be held responsible for those things so to me it's not some big bad threat of being canceled. It's for once powerful people are being held accountable for their actions.

While many respondents, like Jerome, were quick to deny the existence of so-called cancel culture, they tended to also present sometimes conflicting accounts of the importance of public disavowal vis-à-vis accountability for one's actions. What emerged from the data was an additional, inherently complex, theme—contradiction.

A final interpretation we identified among respondents was what we call contradiction—the simultaneous acceptance of multiple, often conflicting, positions as it relates to the legitimacy of so-called cancel culture. While highlighting the nonexclusiveness of the themes discussed, some of the earlier examples documented in this article also demonstrate the theme of contradiction. However, the examples below reflect this theme most clairvoyantly. Several respondents expressed internal or external conflicts about the role of cancel culture in society, the so-called canceling of Glassman, and how this case influenced their own relationship with the sport. Take Britni's response as illustrative of this theme:

*Interviewer*: What's your view of cancel culture?

*Britni* (White, 46, lawyer): Ah … this is a tough question. The lawyer in me has one thought on the matter and the non lawyer in me has another. From the legal perspective cancel culture can be pretty damaging. It can be an issue that we need to deal with legally. On the other hand, people need to be how to a standard especially when they are influential people and authority figures. If you say something ignorant or harmful you should have to face some sort of consequences, and in some situations those consequences include being, you know, canceled, if you want to call it that. And then when you think about it if we live in a world where people have the power to without evidence definitively state something is true about another person that results in them getting so called canceled, isn't that an issue that we have to consider an address. I mean we have courts of law to determine these things, we can't just have the sort of gang mentality on social media that results in potentially really negative outcomes for people who may not have done anything wrong. In the case of Glassman, I don't know that whole ‘Floyd-19’ thing was really gross, and he should face consequences, but in other contexts I can see it being really harmful for society.

As another example, Jason discussed his own internal conflict vis-à-vis cancel culture versus, accountability, and the impacts of public disavowal and renunciation:

Jason: Ahhhh, the Greg Glassman stuff. I think it's more complicated than people think. The guy said some dumb shit. For sure. Like, you can’t read the room man? You know what I mean? But is the guy some massive racist who should get fired and lose his livelihood? I don’t know about that … I’m just not convinced. I just think it's a touchy subject. I remember his Tweet about FLOYD-19 was the biggest thing and yeah that was dumb. The guy needs a PR person to screen his Tweet if he is going to just go all out like that. That is offensive. A guy fucking died, you know? You don’t Tweet that stuff if you’re in a position like that. But the backlash was a bit much to me. Getting rid of the guy, sponsors pulling out of Crossfit games and like even local gyms and stuff. That hurts a lot of people too. Like, some local gyms getting sponsors pulled…that hurts small businesses. I am a small business owner and I’d be pissed if something someone high up in naturopathy Tweeted made some of my partners pull out.

*Ava* (Latin American, 41, occupation unknown): I don’t agree with cancel culture … and what I mean by that is I think there needs to be space for growth and learning … A lot of conflict is that you have a group of people yelling their perspective, you have another group of people yelling their perspective. But there is no conversation and there is no meeting halfway, right? So, then you just have people yelling at each other kind of thing … from my perspective, I think cancel culture isn’t getting us where we need to get to.

Ava went on to tell us:

If (CrossFit) is inclusive and welcoming of different walks of life and stuff like that, then you (Glassman) probably are not the person that needs to be leading that charge right if this is truly what you think behind closed doors kind of thing. So for me, on a personal level, like it wasn’t. I just felt like it [Glassman resigning] was the right thing to happen … like he needed to go.

Jason went even further to avoid characterizing Glassman's comments as racist in the first place while condemning Glassman for not being savvier on social media:

Jason: Would I characterize what Glassman said as racist? Probably not, but it's close. It's a fine line. The goalposts are shifting a lot, you know? The Floyd-19 tweet was pretty bad. I mean … do I think he is a racist for saying something like that, no, but do I think he shouldn’t have tweeted it? Yeah. It was a dumb fucking tweet and people need to realize those things aren’t allowed to be said anymore. It was a dumb tweet and a real insensitive one considering what was happening in the world.

As previously discussed, respondents presenting contradictory perspectives on cancel culture in this case often used the frame of “accountability culture” as a workaround for their own internal understanding of the concept. This sentiment was articulated by Bianca, a Black business student, who coupled her critique of cancel culture with Brock's (2020) notion of “signifyin,” or calling out as a heuristic device rooted in Black vernacular:

*Bianca* (Black, 23, student): You know I don't really like when people call it cancel culture. What happened to Glassman isn't him being canceled … it's him facing the consequences for what he said and what he does. how can I be ‘canceled’? It's not like he didn't deserve it. when you say things in public and when you represent an organization or a company you have to be accountable for the words that you use. They teach us list in first-year business and if you say things that you truly believe but they are harmful for others you have to face the consequences whether that be loss of customers loss of revenue whatever “oh, that's on you.” So, when someone says what happened to Greg Glassman was a result of cancel culture that tells me everything I need to know about the person saying it. to me that makes it seem like that person believes that this was cancel culture and not a logical and rational response to something that was really harmful he said. So, I'm struggling a little bit here because you asked me about cancel culture and I don't necessarily think that this has anything to do with cancel culture. To me cancel culture is when someone who's done nothing wrong is getting called out on social media, not when someone who's done something terrible is held accountable for his actions.

For Bianca, and others, labeling what happened to Glassman as a moment of “cancel culture” is problematic because the label itself assigns a sense of innocence on the perpetrator of harm—as if he was a victim of mob mentality—when, for Bianca, his actions should have legitimate consequences, especially given his position of authority and privilege. Responses like this present a nuanced, albeit sometimes contradictory, approach to the so-called cancel culture—that accountability is vitally important to social life, and one must be responsible for their actions in the public sphere. However, several respondents also indicated that there is a line of accountability in which social pressure—often articulated by references to “mob mentalities” might be counterproductive to the project of keeping people and organizations accountable when they break perceived moral codes. Within the cancel culture continuum, these respondents fall between the affirmation of cancel culture and the rejection of the term and its implications.

## Discussion and Conclusion

It is common today for people to demonstrate their disapproval of others’ behaviors and attitudes through “canceling.” This internet practice, and its associated “cancel culture” is the subject of much debate, with many questioning its efficiency. Emerging research seeks to define cancel culture, trace its histories and etymologies, and understand factors that might influence participation (Brock, 2020; Clark, 2015, 2020; Holman 2020; Nakamura, 2015; Ng, 2020; Skoric et al., 2010). Building on these important contributions, our study examines cancel culture within sporting communities—a prevalent but underresearched phenomenon. As the field currently lacks empirical studies on individual responses, our study used participant interviews to explore how crossfit practitioners interpret and respond to the canceling of an important community figure, Greg Glassman. By locating individual experiences within broader sociological analyses, our research contributes to a holistic understanding of cancel culture. The case of Greg Glassman's “canceling” is particularly insightful given its occurrence within a subculture that is markedly community- and identity-driven, as well as homogeneous and underrepresented by racialized persons. Thus, this study has important theoretical implications for the relationship between cancel culture, identity, community, and race. Our study also provides an alternative perspective to how crossfit practitioners identify with crossfit as a brand and as a sport.

Our analysis of 50 interviews with crossfit practitioners from the Ontario province of Canada revealed a broad continuum that participants responded to cancel culture from affirmation to rejection. Even within these continua of responses, participants were heterogeneous both in their identities and interpretations. These findings support critiques offered by Brock (2020), which challenges the image of “cancelers” as a monolithic mob. Crossfit practitioners in our study offered nuanced responses and displayed critical thinking. In most cases, participants critiqued both Glassman and elements of cancel culture. Even those who were along the rejection end of the cancel culture continuum acknowledged problematic behavior on Glassman's part. The results of our study demonstrate the complex and layered relationships that individuals have with cancel culture, revealing the importance of including such perspectives in future research in the field. We must be wary of oversimplifying community responses to canceling. The cancel culture continuum identified in this article can be used as guidelines in forthcoming work. Potential future areas of study include identifying factors which influence manifold interpretation and the impact of these on behaviors.

Our findings also call into question common stereotypes about crossfit practitioners as evangelical (Beck, 2017) and cult-like (Dawson, 2015; Denby 2013). Participants did not perceive Glassman's beliefs and attitudes as a reflection of their own morals and values, and as such, were able to separate CrossFit the brand from crossfit the sport. For most participants, their identity was not so much attached to the Crossfit brand but to their clubs and practice of the sport. As in the case of Noah, those who had a relationship with the brand were able to let go when they felt it no longer fit within their ideals. These results emphasize the importance of recognizing even the most committed of communities as multidimensional.

Our study also raises important questions as to the role of individual needs and desires in rationalizing canceling choices. Even within participants who affirmed cancel culture, none chose to cancel the sport of crossfit as a whole. How individuals determine what amount of canceling is appropriate to their goals (demonstrating disapproval) is an interesting question for forthcoming research in the field.

Many participants felt that cancel culture was an inappropriate term. Some felt that it had a pejorative connotation used to unfairly critique necessary actions. Others differentiated between “cancel culture” and “accountability culture” and showed a preference for accountability-centered language. It is possible that this disapproval of “cancel culture” is related to what other scholars have identified as a misappropriation of Black practices for highlighting racial inequality (Brock, 2020; Clark, 2015, 2020; Nakamura, 2015). It is difficult to establish this connection given that race was not directly addressed in our interviews. More empirical research is needed to better understand the relationship between race and “cancel culture.” The perceived difference between “canceling” and “holding people accountable” also remains unclear. Future scholarship should address the ways that people differentiate between “cancel culture” and “accountability culture,” and what they perceive to be appropriate measures for holding individuals accountable. It is possible that there is no tangible difference between cancel culture and accountability culture, but participants in our study did perceive a difference. More research could help us understand why and how people differentiate between “cancel” and “accountability” culture, the motivations underlying such perceptions, and whether such perceptions reflect actual differences.

Interestingly, a fear of being canceled was in some of the participants who rejected cancel culture, which included both white and racialized individuals. This was not a fear that participants who affirmed or denied cancel culture expressed. This opens interesting future avenues of inquiry. What factors influence the fear of canceling, and how does this fear relate to affirming, rejecting, or denying cancel culture? Investigating the role of privilege in the fear of cancel culture may provide interesting findings.

Our study sought to better understand individual responses to cancel culture by studying the reactions of crossfit practitioners to Greg Glassman's canceling. We found a conflicted community, whose responses range from affirmation to rejection and denial. These interpretations provide important context for future research in the field. Our results also contradict the generalization of mob mentality within communities experiencing “canceling” and demonstrate an overall dissatisfaction with the language and efficiency of cancel culture. These findings are evidence that more research is needed to understand the complexity of this phenomenon and to develop better strategies for holding others accountable.

## Notes

1. For the purposes of clarity, we must address a key conceptual distinction used throughout this article: when we refer to “CrossFit,” we are referring to the branding organization within the sport and when we use “crossfit” we are referring to the sport itself.
